# Moving targets in 4D-CTs versus MIP and AIP: comparison of patients data to phantom data

**DOI:** 10.1186/s12885-018-4647-4

**Published:** 2018-07-24

**Authors:** Kai Joachim Borm, Markus Oechsner, Moritz Wiegandt, Andreas Hofmeister, Stephanie E. Combs, Marciana Nona Duma

**Affiliations:** 1 Department of Radiation Oncology, Klinikum rechts der Isar, TU München, Ismaninger Str. 22, 81675 Munich, Germany; 2 Medical School Technische Universität München, Ismaninger Str. 22, 81675 Munich, Germany; 3 Institute of Innovative Radiotherapy (iRT), Helmholtz Zentrum München, Ingolstädter Landstraße 1, 85764, Oberschleißheim, Germany

**Keywords:** 4D-CT, MIP, AIP, Moving targets, SBRT

## Abstract

**Purpose:**

Maximum (MIP) and average intensity projection (AIP) CTs allow rapid definition of internal target volumes in a 4D-CT. The purpose of this study was to assess the accuracy of these techniques in a large patient cohort in combination with simulations on a lung phantom.

**Methods:**

4DCT data from a self-developed 3D lung phantom and from 50 patients with lung tumors were analyzed. ITVs were contoured in maximum (ITV_MIP_) and average intensity projection (ITV_AIP_) and subsequently compared to ITVs contoured in 10 phases of a 4D-CT (ITV_10_). In the phantom study additionally a theoretical target volume was calculated for each motion and compared to the contoured volumes.

**Results:**

ITV_10_ overestimated the actual target volume by 9.5% whereas ITV_MIP_ and ITV_AIP_ lead to an underestimation of − 1.8% and − 11.4% in the phantom study. The ITV_MIP_ (ITV_AIP_) was in average − 10.0% (− 18.7%) smaller compared to the ITV_10_. In the patient CTs deviations between ITV_10_ and MIP/AIP were significantly larger (MIP: – 20.2% AIP: -33.7%) compared to this. Tumors adjacent to the chestwall, the mediastinum or the diaphragm showed lower conformity between ITV_10_ and ITV_MIP_ (ITV_AIP_) compared to tumors solely surrounded by lung tissue. Large tumor diameters (> 3.5 cm) and large motion amplitudes (> 1 cm) were associated with lower conformity between intensity projection CTs and ITV_10−_.

**Conclusion:**

The application of MIP and AIP in the clinical practice should not be a standard procedure for every patient, since relevant underestimation of tumor volumes may occur. This is especially true if the tumor borders the mediastinum, the chest wall or the diaphragm and if tumors show a large motion amplitude.

## Background

Radiotherapy is an indispensible part of lung cancer treatment. It is estimated to be necessary in 50% of patients with small cell lung cancer (SCLC) and in over 60% of patients with non-small-cell lung cancer (NSCLC) in the course of the disease [1]. According to several studies, local control rates of over 95% can be achieved using stereotactic body radiotherapy (SBRT) [2, 3]. However, the treatment success hinges on an accurate target volume definition [4]. Target delineation in the lung is especially challenging due to tumor motion caused by respiration. The extent of motion depends on tumor localization and the patient’s breathing pattern. For tumors located close to the diaphragm, amplitudes of over 2.5 cm have been measured [5, 6]. To detect tumor motion accurately, the use of four-dimensional computed tomography (4DCT) is a reliable tool. The 4D-CT generates multiple CT-images, each representing the tumor localization and extent at a certain breathing phase. Contouring of the tumor is usually performed in every single breathing phase with subsequent definition of an internal target volume (ITV) that takes the complete cycle of movement into account. There is good evidence that the use of a 4D-CT reduces motion artifacts and makes target localization more reliable compared to the 3D-CT [7, 8]. This results in better tumor coverage and a decrease of normal tissue irradiation during the treatment [9]. The ITV concept is commonly used for motion management. It ensures excellent tumor coverage but exposes a larger part of healthy lung tissue to radiation. Active motions management such as breathing coordination (gating) and tracking allow smaller treatment volumes and reduction of the dose in the organs at risk (OAR) [10, 11]. However these techniques require information on the tumor position in real time and therefore more complex technical equipment. Thus, the ITV concept remains the preferred motion management technique for many clinics. A major disadvantage of the 4D-CT is the fact that outlining gross tumor volumes (GTVs) in multiple CTs can be time-consuming, especially if a large tumor volume is contoured. Thus, since the introduction of the 4D-CT, alternative contouring methods have been discussed. On the one hand the ITV could be contoured on fewer breathing phases (usually the extreme ones) [9]. On the other hand the ITV might be contoured in average (AIP) or maximum intensity projection (MIP) [12, 13]. Several phantom studies concluded that MIP and AIP are reliable tools for target definition [14, 15]. However there is a lack of clinical studies confirming these findings. A few studies based on small patient collectives (< 20 patients) showed that contouring in MIP might be an adequate option for smaller lung cancers (UICC Stage I) [12, 13, 16]. However a study by Cai et al. [17] using dynamic magnetic resonance imaging as reference concluded that 4D-CT MIP image might cause underdosing due to inaccurate target delineation. Thus the present literature is inconclusive and does not allow any clear conclusions. This study was performed to assess the error of MIP and AIP with special emphasis on tumor localization analyzing a large patient collective in combination with simulations of patient movements on a self-developed lung phantom [18, 19].

## Methods

### Phantom

**Fig. 1:**
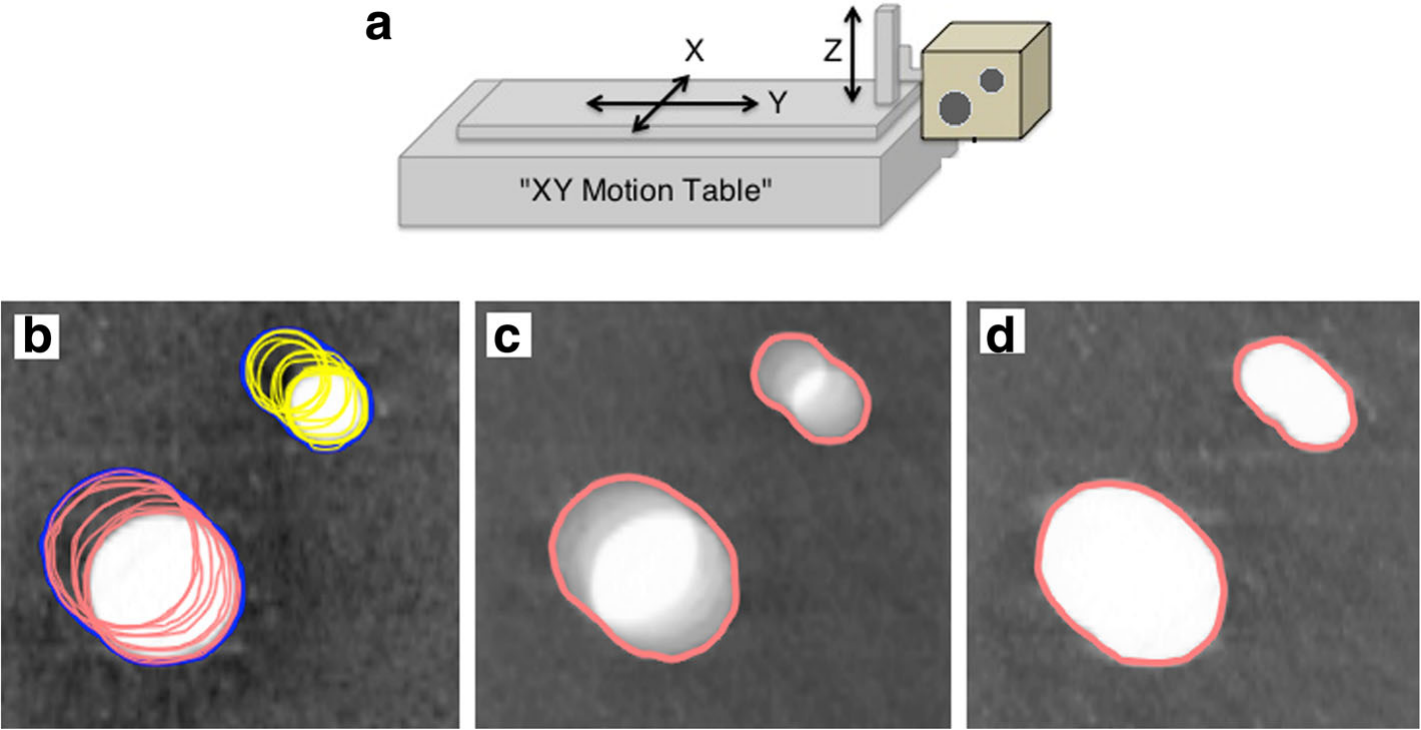
Illustration of the lung phantom (a) and delineation examples of the two targets (Ø 1 cm and Ø 2 cm) in one out of 10 phases of the 4DCT (b), in average intensity projection (c), in maximum intensity projection (d)

A programmable phantom (Fig. 1) was developed based on a xy-table device by SunNuclear® (Sun Nuclear Corporation, Melbourne, FL, USA) that enabled x and y movement. The device was combined with a self-developed equipment that allowed additional movement along the z-axis to simulate 3 dimensional movement. Two spherical structures (Ø 1 cm and Ø 2 cm) composed by water equivalent synthetic substance (RW3) were embedded in corkboards to resemble lung tissue. Tumor movement of 10 patients with large tumor motions (amplitudes > 0.5 cm) out of the patient population described below were reconstructed by using the center of mass motion of the gross tumor volumes delineated in each of the 10 phases of 4D-CT scans. The movement pattern was then simulated with the phantom during CT imaging.

### Patient population

**Fig. 2:**
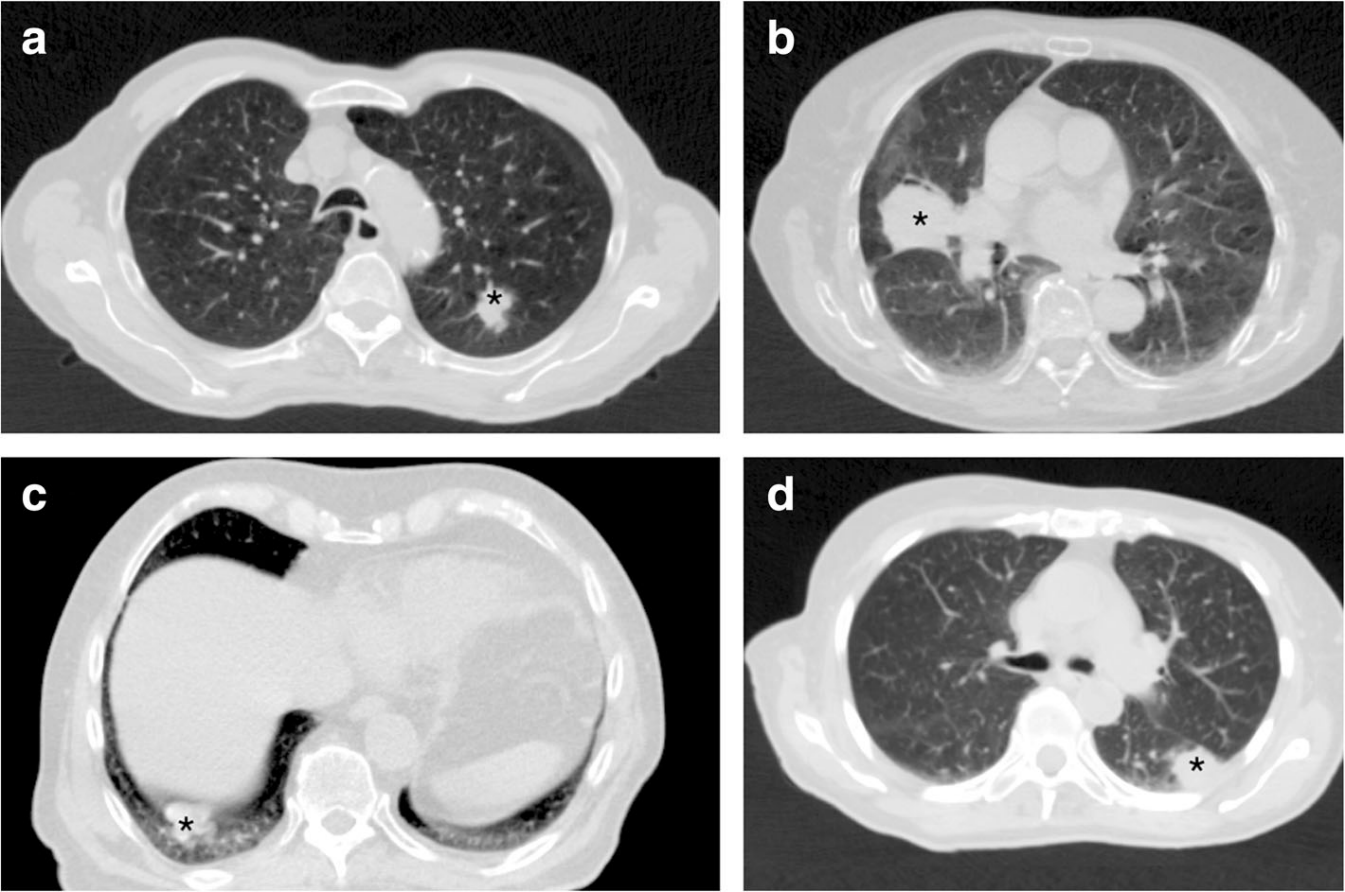
Lung tumors (*) adjoining different structures in the thorax: A) only lung tissue; B) the mediastinum; C) the diaphragm; D) the chest wall

Fifty patients with lung tumors treated with SBRT in our institution were chosen for this study. The diameter of the tumors ranged between 1.1 cm and 7.0 cm; the median value was 3.1 cm. The tumors were distributed in the upper (*n* = 23), middle (*n* = 12) and lower lobe (*n* = 15) of the left (*n* = 22) and right lung (*n* = 28). In 10 cases the tumor was entirely surrounded by lung tissue. The remaining 40 tumors were adjacent to the mediastinum, the chest wall or the diaphragm (Fig. 2). The ethics committee of Klinikum rechts der Isar/Technical University Munich approved this retrospective study. All patients gave their informed consent both informed and written before starting the radiotherapy that they will undergo CT radiotherapy treatment planning. Data from the CT radiotherapy treatment planning were retrospectively analyzed. Immobilization was achieved with a vacuum couch and low pressure foil (Medical Intelligence GmbH, Schwabmünchen, Germany). During irradiation the patients received oxygen supply to further reduce respiratory movement. All plans were calculated for treatment on a Clinac Trilogy linear accelerator equipped with a 120 HD MLC (Varian Medical Systems, Palo Alto, CA, USA).

### 4D-CT data acquisition and contouring

All CT data were acquired using a Somatom Emotion computer tomography (Siemens Medical Solutions, Erlangen, Germany). The breathing curve was detected by the Real-time Position Management system (RPM, Varian Medical Systems, Palo Alto, CA, USA). The spatial and temporal information was used to generate 4D-CTs containing 10 different breathing phases. The same technique was applied both for the lung cancer patients and the moving phantom. Contouring was performed using the Eclipse software Version 13.0 (Varian Medical Systems, Palo Alto, CA, USA). The visible volume of the tumor in the lung or in the phantom, respectively, was contoured slice by slice. [20]

The CT images were viewed and contoured in standard mediastinal and lung window settings (− 125 HU to 225 HU and − 1000 HU and 200 HU). The contouring of the ITVs was performed manually following a visual approach. The same person performed all contouring to avoid interobserver variability [18, 19]. For every structure a gross target volume (GTV) was defined in each of the 10 phases of the 4D-CT. Subsequently an ITV_10_ was generated by merging the 10 GTVs.

Additionally maximum (MIP) and average (AIP) intensity projection CTs were generated based on all phases of the 4D-CT, followed by contouring of an ITV_MIP_ and ITV_AIP_, respectively.

### Analysis of target volumes

For every patient the tumor size and diameter was measured in the treatment planning software. The size of the ITV_10_, ITV_MIP_ and ITV_AIP_ was derived from the treatment planning software after contouring. The average values of ITV_MIP_ and ITV_AIP_ were calculated and compared to the average ITV_10_. Furthermore, to examine the conformity of the generated structures, we measured the overlap (V_OL_) of ITV_10_ and ITV_MIP_ or ITV_AIP_. Subsequently, conformation numbers (CN) were calculated according to van’t Riet et al. [21] as follows: .

$$ CN=\frac{V_{OL}}{ITV_{IP}}\times \frac{V_{OL}}{ITV_{10}} $$

**CN:** conformation number; **V**_**OL**_: overlapping volume between ITV_10_ and ITV_MIP_ or ITV_AIP_; **ITV**_**IP**_: volume of intensity projection (IP) CTs MIP and AIP. **ITV**_**10**_: volume of the target volume based on 10 phases of a 4D-CT.

ITVs measured in the lung phantom (ITV_10_, ITV_MIP_, ITV_AIP_) were additionally compared to mathematically calculated volumes for each motion pattern and tumor diameter. Statistical analysis was performed using SPSS version 24.0 (IBM, Armonk, NY, USA). For all measurements mean values and standard deviation were calculated. The differences between the ITVs (e.g. ITV_MIP_ and ITV_10_) were tested for statistical significance using a two-sided paired T-test. The threshold for statistical significance was set to *p* < 0.05.

## Results

### Lung phantom

For each motion a theoretical ITV was calculated taking the movement pattern and size of the target into account (“theoretical ITV”). This theoretical ITV ranged between 6.7 cm^3^ and 57.5 cm^3^, depending on target motion and size. The average ITV_calc_ for the small target (diameter 1 cm) was 8.6 ± 1.3 cm^3^ and for the large target (diameter 2 cm) 50.3 ± 4.8 cm^3^. The ITV_10_ were significantly larger compared to the calculated values, both for the small (9.3 ± 1.4 cm^3^) as well as for the large target (54.8 ± 4.3 cm^3^). Only in 1 out of 20 cases the ITV_10_ was smaller (− 5,7%) than the calculated values. Contouring in MIP and AIP lead to underestimation of the target volume as compared to the ITV_calc_. 98.1 ± 5.3% and 88.6 ± 5.1% of the calculated values were depicted by MIP and AIP. The ITV_AIP_ showed larger deviations from the calculated values (− 11.4%) than ITV_MIP_ and ITV_10._

**Fig. 3:**
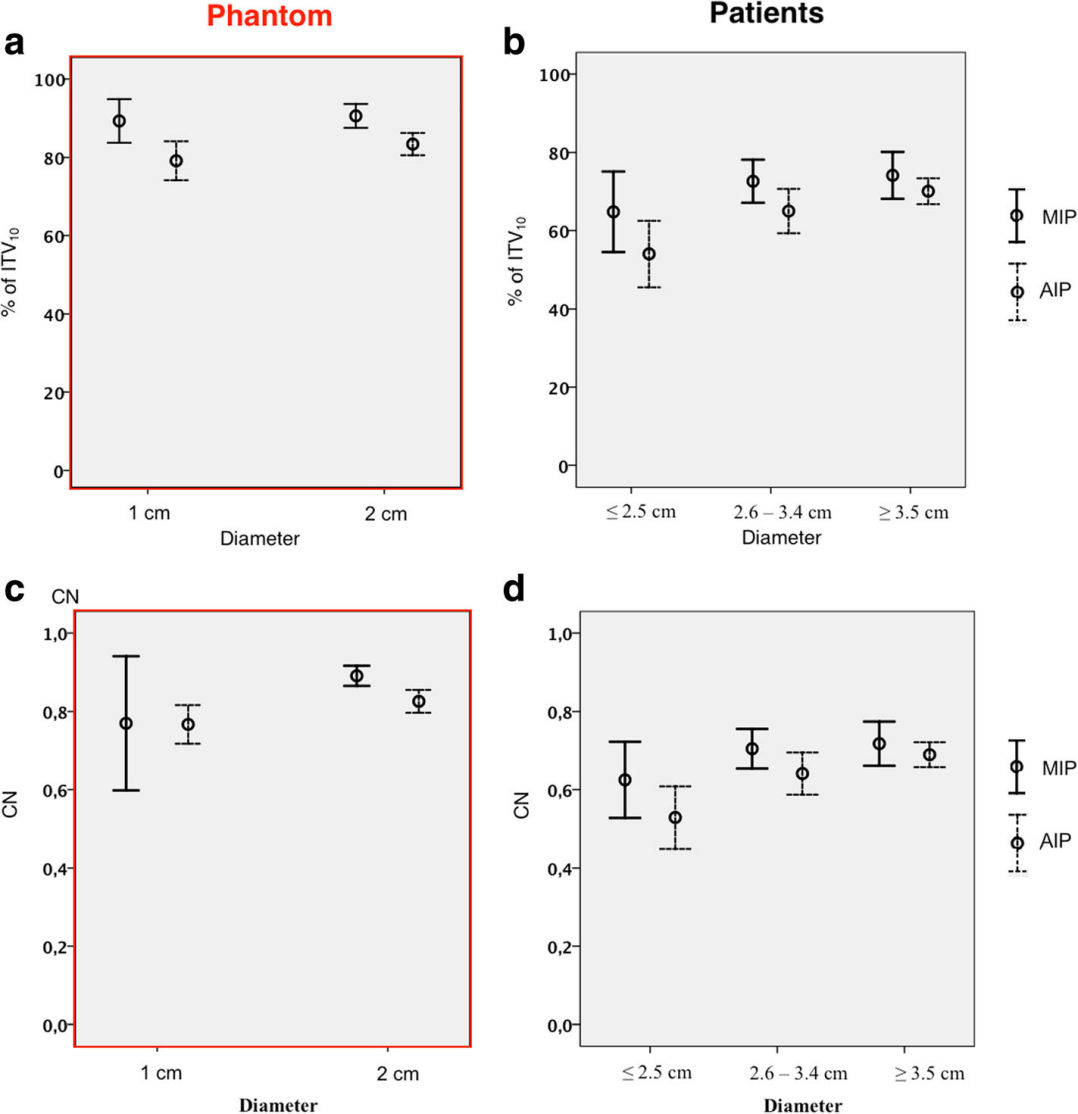
Comparison of values measured in the phantom study **(a,c)** and in the clinical study **(b,d).** Relative size (c,d) and conformation numbers (a,b) of ITVs contoured in MIP and AIP in relation to the ITV_10_

Subsequently ITV_10_, ITV_AIP_ and ITV_MIP_ were compared to each other. Even though ITV_MIP_ and ITV_AIP_ were also significantly smaller compared to the ITV_10_ (ITV_MIP_: -10.0%; ITV_AIP_: -18.7%) in the lung phantom, the differences between the different techniques were significantly smaller compared to the clinical study (Fig. 3). Correspondingly, the phantom conformation numbers between ITV_10_ and ITV_MIP_ or ITV_AIP_ were higher compared to the clinical data (Fig. 3). The mean value for the conformation numbers calculated for the ITV_MIP_ and ITV_AIP_ were 0.83 and 0.79. The ITVs of the Ø 1 cm target showed a larger variability as well as slightly lower conformation numbers compared to the Ø 2 cm target (Table 1).

**Table 1:** Measured values in the lung phantom. Average absolute volumes±SD of the ITV_10,_ ITV_MIP,_ ITV_AIP_ in cm^3^,(relative volumes±SD as percentage of the calculated values -ITV_calc_- in %), conformation numbers (CN) of ITV_MIP_ and ITV_AIP_ with the ITV_10._ n = number of movement patterns

Ø	n=	ITV_calc_	ITV_10_	ITV_MIP_	ITV_AIP_	CN_MIP_	CN_AIP_
1.0 cm	10	8.6 ± 1.3 cm^3^	9.3 ± 1.5 cm^3^ (109.9 ± 13.8%)	8.3 ± 10.9 cm^3^ (97.4 ± 7.1%)	7.3 ± 0.9 cm^3^ (86.2 ± 5.4%)	0.77 ± 0.2	0.77 ± 0.1
2.0 cm	10	50.3 ± 4.8 cm^3^	54.8 ± 4.2 cm^3^ (109.2 ± 5.8%)	49.6 ± 3.8 cm^3^ (98.8 ± 3.0%)	45.7 ± 4.1 cm^3^ (90.9 ± 3.6%)	0.89 ± 0.0	0.89 ± 0.0
Total	20	29.4 ± 21.7 cm^3^	32.1 ± 23.5 cm^3^ (109.5 ± 10.3%)	28.9 ± 21.4 cm^3^ (98.1 ± 5.3%)	26.5 ± 19.9 cm^3^ (88.6 ± 5.1%)	0.79 ± 0.2	0.83 ± 0.1

### Patients

The average amplitude of tumor movement was 0.58 ± 0.35 cm. The mean ITV_10_ of all 50 patients was 27.6 ± 36.3 cm^3^. ITVs based on the MIP or AIP were significantly (*p* < 0.001) smaller compared to the ITV_10_. The ITV_MIP_ had an average value of 21.0 ± 29,7 cm^3^, which corresponded to 73.4 ± 15.6% of the ITV_10._ The ITVs contoured in the AIP CTs were even smaller with an avarage value of 19.2 ± 27.7 cm^3^ (64.8 ± 13.4% of the ITV_10_). The mean overlapping volume of ITV_10_ and ITV_MIP_ was 20.6 ± 29.6 cm^3^. 71.0 ± 13.9% of ITV_10_ were covered in average by the ITV_MIP._ The ITV_AIP_ covered 63.7 ± 12.8% of the ITV_10_ (19.0 ± 27.4 cm^3^). The mean value for the calculated conformity index was 0.69 ± 0.13 in the MIP and 0.63 ± 0.12 in the AIP.

**Fig. 4:**
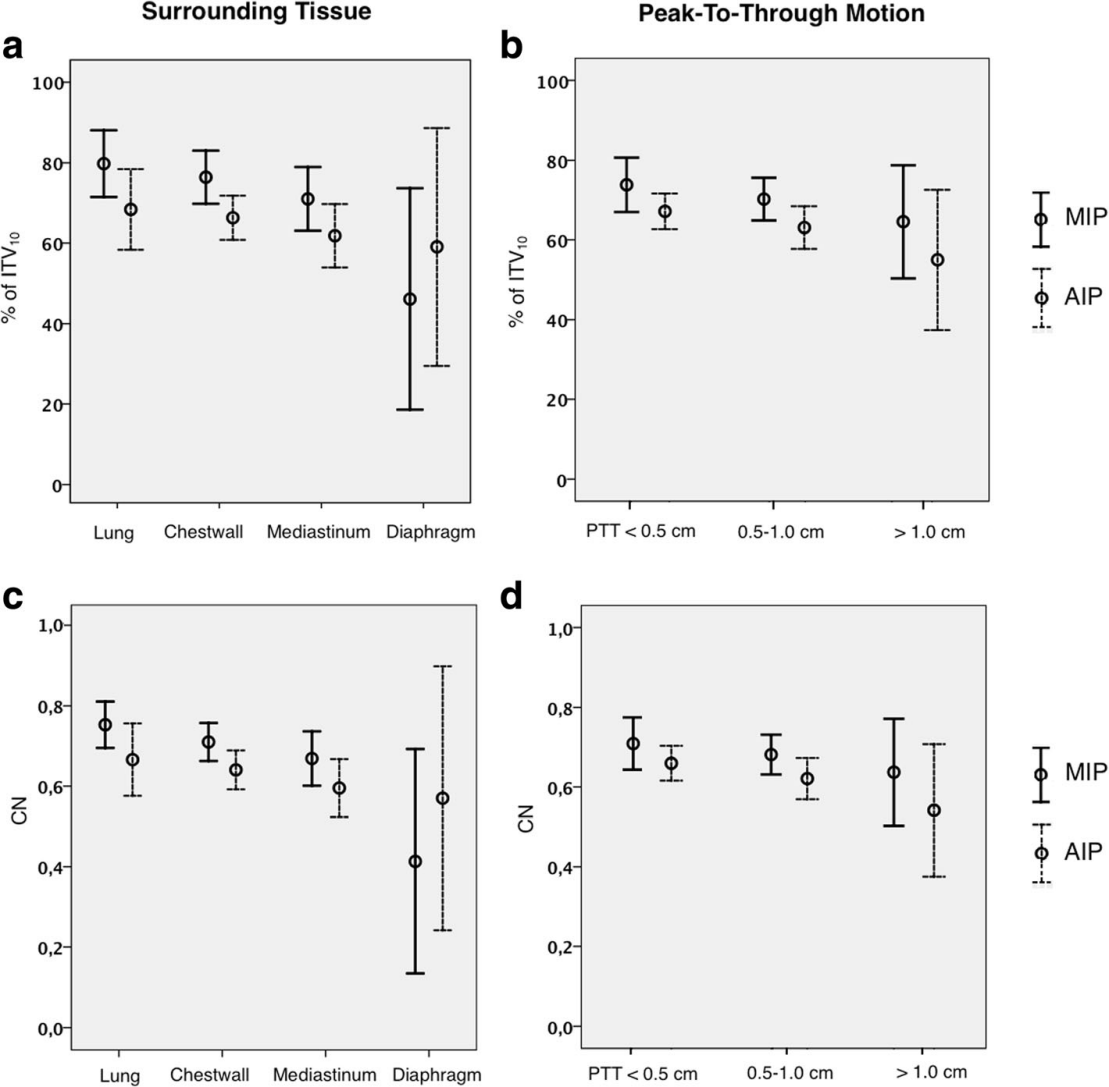
Impact of the surrounding tissue (a,c) and motion amplitude (b,d) on the contoured target volumes in MIP and AIP. Relative size (a,b) in % and conformation numbers (c,d) of ITVs contoured in MIP and AIP in regard to the ITV_10_ CN: conformation number, PTT: peak-to-through motion

Figure 4 depicts the measured target volumes in MIP and AIP considering the surrounding tissue and the tumor movements. MIP and AIP showed the best concordance with the ITV_10_ if the tumor was solely surrounded by lung tissue. For tumors bordering other structures, such as the chest wall or the mediastinum the difference between ITV_10_ and both intensity projection CTs were significantly (< 0.05) larger. The largest deviations between the different contouring techniques (ITV_10,_ ITV_MIP_, ITV_AIP_) were found for tumors merging the diaphragm and abdominal organs (> 50% volume difference). MIP showed better conformity with the ITV_10_ than AIP in all measurements, except for tumors merging to the diaphragm. All values are summarized in Table 2.

**Table 2:** Measured values for ITV_MIP_ and ITV_AIP_ in dependence on the surrounding tissue and the tumor size in a 4D-CT. Relative size in % in regard to the ITV_10_ and overlapping volume (V_OL_) in cm^3^ as well as conformation numbers (CN) of IT V_MIP_ and ITV_AIP_ with the ITV_10_

		n=	MIP			AIP		
			%	V_OL_	CN	%	V_OL_	CN
Tumor surrouding	only lung tissue	10	79.8 ± 11.6	77.5 ± 9.6	0.75 ± 0.1	68.4 ± 14.1	67.5 ± 13.3	0.67 ± 0.1
	chestwall	20	76.4 ± 14.1	73.6 ± 11.3	0.71 ± 0.1	66.3 ± 11.8	65.2 ± 11.0	0.64 ± 0.1
	mediastinum	16	71.0 ± 15.4	68.9 ± 13.9	0.67 ± 0.1	61.8 ± 15.3	60.7 ± 14.5	0.59 ± 0.1
	diaphragm	4	46.1 ± 11.1	43.7 ± 11.1	0.41 ± 0.1	59.1 ± 11.9	58.0 ± 12.4	0.57 ± 0.1
Tumor size (Diameter)	≤2.5 cm	14	67.2 ± 19.4	64.8 ± 17.9	0.63 ± 0.2	55.2 ± 16.0	54.0 ± 14.7	0.53 ± 0.1
	2.6–3.4 cm	19	75.1 ± 13.9	72.6 ± 11.5	0.72 ± 0.1	66.0 ± 12.5	65.0 ± 11.8	0.64 ± 0.1
	≥3.5 cm	17	76.7 ± 13.3	74.1 ± 11.7	0.71 ± 0.1	71.3 ± 6.9	70.1 ± 6.4	0.69 ± 0.1
PTT	< 0.5 cm	21	77.1 ± 16.8	73.8 ± 15.0	0.71 ± 0.1	68.4 ± 10.4	67.2 ± 9.9	0.65 ± 0.1
	0.5–1 cm	22	72.5 ± 13.8	70.2 ± 12.1	0.68 ± 0.1	64.1 ± 12.8	63.1 ± 12.1	0.62 ± 0.1
	> 1 cm	7	65.5 ± 16.3	64.6 ± 15.3	0.63 ± 0.1	55.9 ± 20.2	55.0 ± 19.0	0.54 ± 0.1
Total		50	73.4 ± 15.6	71.0 ± 13.9	0.69 ± 0.1	64.7 ± 13.5	63.7 ± 12.8	0.63 ± 0.1

Also, the tumor size (Fig. 3) and the motion amplitude (Fig. 4) had an influence on the relative differences between ITV_10_ and ITV_MIP_ or ITV_AIP_. For tumors with a diameter of < 2.5 cm only 64.8 ± 17.9% (or 54.0 ± 14.7%) of the ITV_10_ were covered in maximum (or average) intensity projection CTs. Tumors with a diameter that ranged between 2.5 cm and 3.5 cm covered 72.6 ± 11.5% in MIP and 65.0 ± 11.8% in AIP of the ITV_10_. Large tumors (diameter > 3.5 cm) contoured in MIP or AIP (74.1 ± 11.7%; 63.7 ± 12.8%) showed the best conformity to the ITV_10_. Larger tumor amplitudes were associated with poorer conformity between intensity projection CTs and the ITV_10._ The differences between ITV_MIP_ and ITV_AIP_ were 11.6% larger in patients with amplitudes of > 1 cm compared the group with tumor motions of < 0.5 cm (Table 2).

## Discussion

Our results show that ITVs contoured in MIP and AIP differ significantly from ITVs contoured in 10 phases of a 4D-CT. In the clinical study, average deviations of approximately − 25% were observed with even larger differences for tumors that border the mediastinum, the chest wall or the diaphragm. The data acquired with the lung phantom shows that ITV_10_ overestimates the target volume to a certain degree. ITV_MIP_ and ITV_AIP_ on the other hand underestimate the target volume und therefore do not reliably encompass the tumor tissue in all cases. Differences between ITV_10_ and ITV_MIP/AIP_ were substantially larger in the clinical study compared to the phantom study.

A study of Park et al. [14] analyzed the accuracy of MIPs for various target motions using a programmable lung phantom. Two targets inserted in a cork block were moved with irregular target motions along the superior-inferior direction and the two-dimensional target span in moving direction was measured and compared to the theoretical values. They concluded that the MIP accurately reflects the range of motions for regular target motions. However the validity of the results for the clinical practice is limited, since target motion was simulated in only one direction, no volume assessment was performed and tumors in patients seldom undergo a regular movement pattern. Simon et al. [15] used a lung tumor phantom to simulate anterior-posterior movements to compare AIP and MIP ITVs to calculated theoretical values. The error on volume assessment ranged from.

− 40% to − 9% for the AIP and from − 3 to 12% for the MIP. The average deviations from the calculated values measured in our study in the lung phantom were also within this range (Table 1). The authors concluded that MIPs could be used for target definition of moving targets in a 4D-CT, as it seems to encompass the tumor movement. However, before this conclusion is drawn, the question must be raised whether these results from idealized phantom conditions can be transferred to the clinical situation where serrated tumor shapes, complex tumor movements and irregular density distributions occur.

The available clinical date is inconclusive and based on small groups of patients only. Bradley et al. [12] compared MIP, AIP and helical 4D-CT images of 20 inoperable peripheral stage I lung tumors to determine the best definition method for stereotactic body radiation therapy. MIP-defined ITVs were significantly larger than helical and AIP defined ITVs. They concluded MIP is superior to AIP in order to depict tumor motion. However, since no comparison to the ITV_10_ was done, the question whether the actual tumor is represented accurately by the ITV_MIP_ remains unclear in this study. A study by Murihead et al. [13] collected 4D-CT data from 14 patients with NSCLC. ITVs were contoured in 10 phases of a 4D-CT and in MIP. The ITV_10_ served as a reference volume to evaluate the precision of the MIP. In average 19% of the ITV_10_ were not enclosed by the ITV_MIP._ This is in accordance with our findings showing an insufficient coverage of the ITV_10_ in MIP and AIP. The authors proposed the use of the MIP image target delineation for patients with stage I disease, since only minor deviations (6.1%) occurred in this subgroup, which consisted of 2 patients in the study. Contrary to this, in our study small targets (Ø < 2.5 cm in the clinical study, Ø 1 cm in the phantom study) resulted in the poorest conformity between MIP and the ITV_10_ (Fig. 3). Underberg et al. [16] analyzed 4D-CT data from a phantom and from 11 patients with small Stage I lung cancer. ITVs generated in all 10 phases were compared with ITVs generated in MIP. The average ratio between ITV_10_ and ITV_MIP_ was 1.04 for the phantom study and 1.07 in scans of the patients. The center of mass differed by only 0.4 or 0.5 mm, respectively. The authors concluded that MIPs are a reliable clinical tool for generating ITVs from a 4DCT data set. Even though not explicitly mentioned by the authors it appears in the figures that narrow window settings (e.g. mediastinal window) have been used for contouring. As shown in current literature these windows do not accurately reflect moving targets and might have a major effect on the results [18] .

According to our results MIP does not accurately depict the target volume as contoured in each of the 10 phases of a 4D-CT. The deviations between ITV_10_ and ITV_MIP/AIP_ in the clinical study (MIP: – 20.2% AIP: -33.7%) were almost twice as large as in the phantom study (MIP –10.0% AIP -18.7%). Even though the MIP reflected the calculated values in the phantom study well, relevant underestimation of the target size needs to be expected in the clinical practice. This is especially true if the tumor borders the mediastinum, the chest wall or the diaphragm and if tumors show an extensive motion amplitude. For these tumors the deviations were particularly large. The reason for this is a loss of contrast between tumor and surrounding tissue by using maximum values for every voxel, leading to underestimation of the tumor in the overlapping areas. The large deviations using MIP for tumors bordering soft tissues could be also observer dependent, in particular as a visual approach was followed instead of automatic contouring. Since extreme movement of tumors bordering soft tissue impedes definition of an ITV, other treatment options like robotic radio surgery or breath hold techniques should be taken into consideration, in these situations [22, 23].

The 4D-CT has been adopted as a standard modality for target delineation in lung tumors because it represents moving targets significantly better than slow 3D-CTs. Nakamuru et al. [7] evaluated in 32 lung cancer patients the geometrical differences in target volumes between slow CT- and 4D CT- imaging for lung tumors. They observed that target volumes acquired in slow 3D-CTs are approximately 25% smaller compared to target volumes contoured in a 4D-CT. In our study MIP ITVs were on average 20.2% smaller than the ITV_10_, which, by extrapolation, can be compared to the previous reported difference between the slow 3D-CTs and ITV_10._ Thus, the use of MIP comes with a risk of losing additional and relevant information obtained by analyzing all phases of the 4D-CT.

The current study focuses solely on the impact of MIP and AIP on definition of the ITV. However, it needs to be also taken into account that the data set used for treatment planning has an important effect on the dose distribution within the tumor and the OAR [20, 24]. Due to respiration-induced density variations within the ITV 3D, dose calculation based on free-breating-, MIP-, AIP- or mid-ventilation CT datasets only estimates the actual dose in the tumor [10]. The dosimetric characteristics of plans based on AIP and mid-ventilation CTs are reported to be similar to those of FB- CTs [25]. Treatment plans calculated on a MIP CT dataset on the other hand may not be not appropriate for OAR dose assessment [20]. A promising approach to cope with density variations is the use of 4D-CT treatment planning with respiration-correlated assignment of the treatment plan’s monitor units to the different respiration phases of a 4D-CT and subsequent rigid and non-rigid registration [10].

A potential limitation of this study is the impact of interobserver variability on the contouring of lung tumors in a 4D-CT. Louie et al. [26] showed that the percentage shared internal target volume of 6 physicians contouring 10 different tumors ranged from 31.1 to 83.3%. Therefore the observed effect might differ in some cases. Nevertheless we cannot recommend the MIP (and AIP) as a standard procedure in clinical practice, since relevant underestimation of target motion and tumor volumes may occur. Whenever MIP is used for contouring, we strongly recommend to double check that the ITV encompasses the delineated target in each of the 10 phase of the 4D-CT.

## Conclusion

Contouring in maximum or average intensity projection CTs reduces the time that is required to define an internal target volume. Even though the MIP reflected the calculated values in the phantom study well, relevant underestimation of the target size can be expected in the clinical practice. This is especially true if the tumor borders the mediastinum, the chest wall or the diaphragm and if tumors show a large motion amplitude. Therefore neither AIP nor MIP can be unquestionably recommended for target delineation. Whenever MIP is used for contouring, it needs to ensure that ITV encompasses the delineated target in every phase of the 4D-CT.

## Abbreviations

4DCT: Four-dimensional computed tomography
AIP: Average intensity projection
CN: Conformation numbers
GTVs: Gross tumor volumes
HU: Hounsfield units
ITV: Internal target volume
ITV_10_: ITV based on 10 4DCT phases
ITV_AIP_: ITV based on AIP
ITV_calc_: Theoretical calculated ITV
ITV_MIP_: ITV based on MIP
MIP: Maximum intensity projection
NSCLC: Non-small-cell lung cancer
OAR: Organs at risk
SBRT: Stereotactic body radiotherapy
SCLC: Small cell lung cancer
V_OL_: Overlapping volume
